# Determinants of Malnutrition and Post-operative Complications in Hospitalized Surgical Patients

**Published:** 2014-09

**Authors:** Vânia Aparecida Leandro-Merhi, José Luiz Braga de Aquino

**Affiliations:** ^1^School of Nutrition, Clinical Nutrition, PUC-Campinas-SP, Brazil; ^2^School of Medicine, Surgery Department, PUC-Campinas-SP, Brazil

**Keywords:** Complications, Hospitalized surgical patients, Malnutrition, Nutritional status, Brazil

## Abstract

The study aimed to determine the nutritional status (NS) of hospitalized surgical patients and investigate a possible association between NS and type of disease, type of surgery and post-operative complications. The gender, age, disease, surgery, complications, length of hospital stay, number of medications, laboratory test results, and energy intake of 388 hospitalized surgical patients were recorded. NS was determined by classical anthropometry. The inclusion criteria were: nutritional status assessment done within the first 24 hours of admission, age ≥20 years, and complete medical history. Univariate and multiple Cox's regression analyses were employed to determine which variables were possible risk factors of malnutrition and complications. Malnutrition was more common in males (p=0.017), individuals aged 70 to 79 years (p=0.000), and individuals with neoplasms and digestive tract diseases (p=0.000). Malnourished individuals had longer hospital stays (p=0.013) and required more medications (p=0.001). The risk of malnutrition was associated with age and disease. Individuals aged 70 years or more had a two-fold increased risk of malnutrition (p=0.014; RR=2.207; 95% CI 1.169-4.165); those with neoplasms (p=0.008; RR=14.950; 95% CI 2.011-111.151) and those having digestive tract diseases (p=0.009; RR=14.826; 95% CI 1.939-113.362) had a 14-fold increased risk of malnutrition. Complications prevailed in older individuals (p=0.016), individuals with longer hospital stays (p=0.007), and individuals who died (p=0.002). The risk of complications was associated with age and BMI. In the present study, the risk of malnutrition was associated with age and type of disease; old age and low BMI may increase complications.

## INTRODUCTION

The nutritional status of adult and elderly hospitalized patients has been discussed for years. The rates of malnutrition in this population usually depend on disease and assessment criteria and vary from 10% to 50% (1-3). However, the risk of malnutrition varied from 19% to 60% according to a British study (4), was 27.4% according to a German study (5), and 46% according to a Canadian study (6), Finally, a study in Spain found mild, moderate and severe malnutrition rates of 50.7%, 26.4%, and 5.7% respectively (7).

Recent studies in Brazil (8) found a malnutrition rate of 14.1% shortly after admission to hospital. These rates varied according to the assessment method.

Different parameters are being developed to assess the nutritional status of hospitalized patients and better map this reality (5-9). Nevertheless, malnutrition is still underreported (10), despite its association with increased morbidity, mortality, and hospital costs (10).

Malnutrition increases the risk of complications from abdominal surgery (11,12) but weight loss, low albumin, and low body mass index (BMI) are not always associated with mortality and morbidity in surgical patients (13). Although many studies have assessed the nutritional status of hospitalized patients, including some from this research group (8,14,15), the relationship between nutritional status and other variables, such as type of disease, type of surgery, and occurrence of complications, among others, should be further explored. Newfound Associations may help improve interventional actions and control strategies that aim to prevent malnutrition-related intercurrences.

The objective of this study was to determine the nutritional status of hospitalized surgical patients and investigate whether their nutritional status was associated with type of disease, type of surgery, and post-operative complications.

## MATERIALS AND METHODS

This study was conducted at the university hospital (*Hospital e Maternidade Celso Pierro*) of the Pontifical Catholic University of Campinas, a large university in the state of São Paulo, Brazil, from 2010 to 2011, after approval from the local Research Ethics Committee. This university hospital is a tertiary-level hospital that routinely treats high-risk patients, such as those with polytrauma, and performs complex surgeries for cancer. Its catchment areas are the city of Campinas and the respective metropolitan regions.

The study is part of a research project called “Nutritional status of hospitalized patients and its relationship with disease, clinical and surgical variables, and length of hospital stay.” Since the study location was the surgical ward, the study patients were surgical patients. The inclusion criteria were: nutritional status assessed within the first 24 hours of admission, age ≥20 years, and availability of complete medical records. The exclusion criteria were: terminal patients, patients with oedema or ascites, patients undergoing haemodialysis, patients with psychiatric diseases, patients kept in isolation, patients of ocular surgery, and those admitted only for clinical investigation and/or tests. Bed-ridden patients or patients who could not talk were also excluded since their body-weight and habitual energy intake (HEI) could not be determined. At first, 512 adult and elderly patients (aged >60 years according to the Brazilian Elderly Statute) in the surgical ward were selected systematically but, after applying the selection criteria, 388 retained, constituting the final sample.

### Data collection

A protocol was developed specifically for this study to collect the following data systematically from the patients’ medical records during their stay: gender, age, length of stay (LOS) at the hospital, type of disease, type of surgery, post-operative complications, anthropometric indicators of nutritional status, laboratory test results, HEI, and number of medications prescribed during the stay.

### Nutritional status assessment

Body-weight, height, arm-circumference (AC), triceps skinfold thickness (TST), and calf-circumference (CC) were measured; and body mass index (BMI), arm muscle-circumference (AMC), arm muscle-area (AMA), and arm fat-area (AFA) were then calculated. The patients were also asked whether they had gained, maintained, or lost weight in the six months before admission, and their weight changes were classified accordingly.

The BMIs of adults aged <60 years were calculated and classified as recommended by the World Health Organization (16) and those of the elderly people (≥60 years of age) as recommended by Lipschitz (17).

The parameters AC, AMC, AMA, TST, and AFA of adults aged ≤65 and >65 years were classified according to the percentile distribution reference values given by Frisancho (18) and Burr and Phillips (19) respectively. Patients were considered to be wasting when their AC, AMC, and AMA were equal to or below the 5th percentile (≤P5); at risk of wasting when those parameters were between the 5th and 15th percentiles (P5-P15); and with preserved lean body mass (PLBM) when those parameters were above the 15th percentile (>P15). Fat mass was considered depleted (DFBM) when TST and AFA were equal to or below the 5th percentile (≤P5); at risk of depletion (RDFBM) when those parameters were between the 5th and 15th percentiles (P5-P15); and preserved lean body mass (PFBM) when those parameters were above the 15th percentile (>P15) (18,19). Only the elderly's CCs were measured and classified as recommended by the WHO (20), using the cutoff point of 31 cm.

### Habitual energy intake (HEI) assessment

The patients were interviewed individually to determine habitual food intake. The software NutWin® (2002) (21) was then used for calculating energy intake. The percentage of HEI adequacy (% HEI/ER) was calculated for each individual. Individual requirements were estimated by the Harris and Benedict equation (22) as described elsewhere (8,14). Energy intake was considered low when it was <75% of the individual's requirement (HEI/ER <75%) (23,24).

### Variable classification

The diseases were classified as follows: digestive tract diseases (peptic ulcers, bowel diseases, inflammatory bowel diseases, pancreatitis, gall bladder diseases, and others), gynaecological diseases (endometriosis, ovary cysts, and others), vascular diseases (peripheral artery diseases, aneurisms), neoplasms (malignant neoplasms), and trauma (polytrauma). Types of surgery were classified as head and neck surgery, digestive system surgery, gynaecological surgery, orthopaedic surgery, plastic surgery, thoracic surgery, urologic surgery, vascular surgery, neurosurgery, and exploratory laparotomy. Complications were defined as clinical intercurrences that occurred after surgery and classified as cardiovascular, infectious, pulmonary, other, and no complications. Laboratory tests included that for haemoglobin and lymphocyte counts, and both were considered risk factors when found below the reference range (25).

### Definition of malnutrition

The diagnosis of malnutrition (on admission) was based on the assessments of anthropometric indicators. Individuals were considered malnourished when BMI was <18.5 kg/m^2^ for adults and ≤22 kg/m^2^ for the elderly; or BMI <20.0 kg/m^2^ and AMC or TST equal to or below the 15th percentile (≤P15) (2,26).

### Study of associated factors

All the anthropometric and laboratory variables, HEI, LOS, gender, age, type of the disease, type of surgery, and number of medications prescribed during hospital stay were tested for association with malnutrition and complications. The following were considered possible risk factors of malnutrition: gender, age, disease, HEI, and low haemoglobin count (lymphocyte count was not included in multiple analyses because of limited information). The following were considered possible risk factors of complications: gender, age, disease, malnutrition, anthropometric variables, HEI, low haemoglobin (again, lymphocyte count was not included for the same reason mentioned above), and number of medications prescribed during stay at the hospital.

### Statistical analyses

The chi-square test or Fisher's exact test were used for verifying associations or comparing proportions (for gender, age-group, type of disease, type of complications, type of surgery, anthropometric indicators, energy intake, length of stay at the hospital, and outcome, i.e. death or discharge).

Continuous or ordinal measures between two groups were compared by the Mann-Whitney test. The risk factors of malnutrition and complications were determined by Cox's regression. The relative risk (RR) and respective confidence intervals (CIs) of 95% were also calculated (27,28). A univariate regression analysis of each factor of interest was done, followed by multiple regression analyses. Variables were selected by the stepwise method. The significance level was set at 5% (p<0.05). The data were treated by the software SAS (Statistical Analysis System) (29).

## RESULTS

The sample consisted of 388 patients: 204 (52.58%) females and 184 (47.42%) males; 167 (43.04%) stayed at the hospital for up to 3 days; 122 (31.44%) stayed for 4 to 7 days; and 99 (25.52%) stayed for 8 days or more. Ten (2.58%) patients died. The rate of malnutrition was 15.98%. The rate of malnutrition dropped to 12.37% if only BMI was used. Almost half of the sample (42.97%) had an HEI/ER <75%; 20.77% had lost weight recently; and 43.04% had low haemoglobin level.

Comparison of nourished (N=326) and malnourished (N=62) patients showed that malnutrition was more prevalent in males, individuals aged 70 to 79 years, individuals with neoplasms or digestive tract diseases, and individuals subjected to digestive system or head and neck surgery (Table 1). As a matter of fact, individuals admitted for head and neck surgery were already more malnourished at admission. Table 1 also shows that complications were more common in older individuals, those staying at the hospital for ≥7 days, and individuals who died. Individuals subjected to digestive tract surgery or with neoplasms also tended to have complications but the difference was not significant. More information can be found in Table 1.

Malnourished individuals had significantly lower AC, TST, AMC, AMA, and CC. The CC was a good predictor of malnutrition in the elderly. Recent weight loss was also associated with malnutrition as well as stay at the hospital for >7 days. AFA, low haemoglobin count, HEI/ER <75%, and death were not associated with malnutrition. Not all the individuals who died were malnourished (Table 2). Table 3 shows the comparison between other variables of the malnourished and nourished groups. Age, LOS, and lymphocyte count differed significantly between the groups. Malnourished individuals were older, had longer LOS, were prescribed more drugs during their stay at the hospital, and had lower lymphocyte counts. Significant differences were also found between some variables of the groups with and without complications, namely age, LOS, and haemoglobin level (Table 3).

**Table 1.** Comparison of the study variables of the nourished and malnourished groups and the groups with and without complications

Variable	Nourished n (%)	Malnourished n (%)	p value	No complication n (%)	With complication n (%)	p value
Females	180 (55.21)	24 (38.71)	0.0170*	169 (52.3)	35 (53.8)	0.8223*
Males	146 (44.79)	38 (61.29)		154 (47.7)	30 (46.1)	
Age (completed years)						
<60	228 (69.94)	31 (50.0)	0.0007*	226 (69.9)	33 (50.8)	0.0167*
60 to 69	53 (16.26)	10 (16.13)		49 (15.1)	14 (**21.5)**	
70 to 79	31 (9.51)	17 (**27.42)**		36 (11.1)	12 (**18.5)**	
≥80	14 (4.29)	4 (6.45)		12 (3.7)	6 (**9.2)**	
Type of disease						
Digestive tract	63 (19.33)	16 (**25.81)**	0.0001*	66 (20.4)	13 (20.0)	0.1664*
Gynaecological	84 (25.77)	4 (6.45)		78 (24.1)	10 (15.4)	
Vascular	43 (13.19)	5 (8.06)		41 (12.7)	7 (10.8)	
Neoplasms	87 (26.69)	32 (**51.61)**		91 (28.2)	28 (43.1)	
Trauma	49 (15.03)	5 (8.06)		47 (14.6)	7 (10.8)	
Type of surgery						
Head and neck	24 (7.36)	12 (**19.35)**	0.0018**	29 (8.9)	7 (10.8)	0.7176**
Digestive system	82 (25.15)	23 (**37.10)**		82 (25.4)	23 (35.4)	
Gynaecological	67 (20.55)	6 (9.68)		62 (19.2)	11 (16.9)	
Orthopaedic	37 (11.35)	-		34 (10.5)	3 (4.6)	
Plastic	10 (3.07)	-		8 (2.5)	2 (3.1)	
Thoracic	5 (1.53)	-		5 (1.6)	-	
Urologic	26 (7.98)	5 (8.06)		27 (8.4)	4 (6.1)	
Vascular	29 (8.90)	6 (9.68)		29 (8.9)	6 (9.2)	
Neurosurgery	18 (5.52)	3 (4.84)		19 (5.9)	2 (3.1)	
Laparotomy	28 (8.59)	7 (**11.29)**		28 (8.7)	7 (10.8)	
Complications						
Yes	52 (15.95)	13 (20.97)	0.3322*			
No	274 (84.05)	49 (79.03)				
Type						
Cardiovascular	36 (11.04)	4 (6.45)	0.0964**			
Infectious	12 (3.68)	5 (8.06)				
Pulmonary	1 (0.31)	3 (4.84)				
Other	3 (0.92)	1 (1.61)				
No complication	274 (84.05)	49 (79.03)				
LOS						
Up to 6 days				229 (70.9)	35 (53.8)	0.0071*
≥7 days				94 (29.1)	30 (46.1**)**	
Death						
Yes				4 (1.2)	6 (9.2)	0.0022**
No				319 (98.8)	59 (90.8)	

Laparotomy=Exploratory laparotomy; Type=Type of complication; LOS=Length of stay at hospital;

*Chi-square test;

**Fisher's exact test

**Table 2.** Comparison of the categorical variables of the nourished and malnourished groups

Nutritional indicator	Classification	Nourished n (%)	Malnourished n (%)	p value
Arm-circumference	≤P5	22 (6.8)	31 (50.8)	<0.0001*
	P5-P15	42 (12.9)	13 (21.3)	
	>P15	260 (80.2)	17 (27.8)	
Triceps skinfold thickness	≤P5	9 (2.8)	9 (14.8)	<0.0001**
	P5-P15	17 (5.3)	14 (22.9)	
	>P15	297 (91.9)	38 (62.3)	
Arm muscle-circumference	≤P5	55 (17.0)	34 (56.7)	<0.0001*
	P5-P15	56 (17.3)	7 (11.7)	
	>P15	211 (65.5)	19 (31.7)	
Arm muscle-area	≤P5	50 (15.6)	30 (50.8)	<0.0001*
	P5-P15	35 (10.9)	11 (18.6)	
	>P15	236 (73.5)	18 (30.5)	
Arm fat-area	≤P5	25 (9.0)	8 (17.8)	0.0692**
	P5-P15	9 (3.2)	3 (6.7)	
	>P15	241 (87.6)	34 (75.5)	
Calf-circumference***	≥31 cm	55 (63.2)	4 (15.3)	<0.0001*
	<31 cm	32 (36.8)	22 (84.6)	
Haemoglobin level	No risk	152 (58.9)	24 (47.0)	0.1182**
	At risk	106 (41.0)	27 (52.9)	
Recent weight change	Weight gain	70 (22.9)	12 (20.0)	0.0010*
	No change	183 (59.8)	25 (41.7)	
	Weight loss	53 (17.3)	23 (38.3)	
HEI/ER <75%	No	186 (58.5)	29 (49.1)	0.1833*
	Yes	132 (41.5)	30 (50.8)	
Length of stay at hospital	Up to 6 days	230 (70.5)	34 (54.9)	0.0150*
	≥7 days	96 (29.4)	28 (45.2)	
Deceased	Yes	6 (1.8)	4 (6.4)	0.0587**
	No	320 (98.1)	58 (93.5)	

*Chi-square test;

**Fisher's exact test;

***Only in elderly patients; HEI/ER <75%=Habitual energy intake <75% of the energy requirement

Univariate Cox's regression was used for identifying the risk factors of malnutrition, followed by multiple analysis with the variables, such as gender, age, disease, haemoglobin level, and HEI/ER <75%─all selected by the stepwise method. Table 4 shows the model that best predicted malnutrition. The rate of malnutrition in the category ‘gynaecological diseases’ was low (6.4%) (Table 1). So, this category was used as reference for comparison with other disease categories and possible risk factors of malnutrition. Risk of malnutrition was associated with age and type of the disease. Patients aged 70 years or more had a two-fold increased risk of malnutrition, and patients with neoplasms or digestive tract diseases had a 14-fold increased risk of malnutrition. Hence, age and type of disease were the main risk factors of malnutrition (Table 4).

Body composition indicators, BMI, recent weight change, HEI/ER <75%, haemoglobin level, and degree of malnutrition did not differ between the group of patients that had complications and the group that did not have complications.

Table 5 shows the model that best predicts complications (univariate analysis followed by multiple Cox's regression with the variables selected by the stepwise method). Risk of complications was associated with age and BMI. Each year of life and each additional BMI integer increased the risk of complications by 1.03 and 1.07 respectively (Table 5).

**Table 3.** Comparison of the numerical variables of the nourished and malnourished groups and of the groups with and without complications

Study variable	N	Mean±SD	Median	p value*
Age (years)				
Nourished	326	49.9±16.9	50.0	0.0044
Malnourished	62	56.4±18.5	59.0	
No complications	323	49.5±17.1	50.0	0.0002
With complications	65	58.4±16.4	59.0	
LOS (days)				
Nourished	326	5.9±6.0	4.0	0.0132
Malnourished	62	8.1±8.6	6.0	
No complications	323	5.6±5.1	4.0	<0.0001
With complications	65	9.4±9.3	6.0	
HEI (kcal)				
Nourished	321	1,758±701.3	1,600.3	0.0933
Malnourished	59	1,576±562.7	1,438.1	
No complications	318	1,756.1±707.8	1,580.0	0.2076
With complications	62	1,593.9±531.1	1,579.2	
TER (kcal)				
Nourished	323	2,088±367.5	2,027.8	0.1878
Malnourished	62	2,021±384.2	1,977.3	
No complications	320	2,079.9±373.9	2,010.1	0.8660
With complications	65	2,066.2±355.70	2,025.24	
HEI/ER <75%				
Nourished	318	85.2±33.2	78.8	0.2751
Malnourished	59	80.9±31.7	72.7	
No complications	315	85.5±33.7	78.7	0.3345
With complications	62	80.0±28.6	75.6	
Number of prescriptions				
Nourished	259	5.9±3.5	5.0	0.0017
Malnourished	50	7.4±3.6	7.0	
No complications	260	6.1±3.6	5.0	0.7704
With complications	49	6.2±3.2	5.0	
Haemoglobin level				
Nourished	258	12.8±2.9	13.1	0.2418
Malnourished	51	12.2±2.6	12.6	
No complications	251	12.9±3.0	13.1	0.0379
With complications	58	12.1±2.5	12.2	
Lymphocyte count				
Nourished	145	1,859±1171.8	1,680.0	0.0159
Malnourished	34	1,427±723.0	1,202.0	
No complications	139	1,784.5±1127.1	1,642.0	0.7409
With complications	40	1,754.2±1071.6	1,580.5	

*Mann-Whitney test; HEI=Habitual energy intake; HEI/ER <75%=Habitual energy intake <75% of the energy requirement; LOS=Length of hospital stay; TER=Total energy requirement

## DISCUSSION

This work was part of another research that studied the nutritional status of hospitalized surgical patients (8,14,15). Assessment of 388 patients found that 15.9% were malnourished, 20.7% had lost weight in the 6 months before admission, and 42.9% had HEI/ER <75%. Hence, a considerable proportion of this population could be considered at risk of malnutrition shortly after admission. These findings corroborated those from other studies (2,5,6). Additionally, more than 10% of the sample presented with wasting or fat mass depletion.

**Table 4.** Risk factors associated with malnutrition according to univariate and multiple Cox's regression

Univariate analysis				
Variable	Reference	p value	Relative risk	CI (95%)
Gender	Male vs Female	0.0309	1.755	1.053-2.926
Age-group	60-69 vs <60 years	0.4376	1.326	0.650-2.705
Age-group	≥70 vs <60 years	0.0005	2.658	1.528-4.626
Age		0.0145	1.019	1.004-1.034
Disease	DTD vs Gynaecological	0.0075	4.456	1.490-13.328
Disease	Vascular vs Gynaecological	0.2164	2.292	0.615-8.534
Disease	Neoplasms vs Gynaecological	0.0008	5.916	2.092-16.728
Disease	Trauma vs Gynaecological	0.2889	2.037	0.547-7.586
AC	≤P5 vs >P15	<0.0001	9.529	5.274-17.217
AC	P5-P15 vs >P15	0.0003	3.852	1.871-7.930
AMA	≤P5 vs >P15	<0.0001	5.292	2.950-9.492
AMA	P5-P15 vs >P15	0.0015	3.375	1.594-7.146
AFA	≤P5 vs >P15	0.0866	1.961	0.908-4.236
AFA	P5-P15 vs >P15	0.2423	2.022	0.621-6.584
Haemoglobin		0.2204	0.937	0.845-1.040
HEI/ER <75%		0.3993	0.996	0.988-1.005
Lymphocytes		0.0521	1.000	0.999-1.000
Multiple analysis			
n=48 vs n=252				
Age-group	60-69 vs <60 years	0.5814	1.247	0.569-2.733
Age-group	≥70 vs <60 years	0.0146	2.207	1.169-4.165
Disease	DTD vs Gynaecological	0.0094	14.826	1.939-113.362
Disease	Vascular vs Gynaecological	0.0568	8.103	0.941-69.753
Disease	Neoplasms vs Gynaecological	0.0082	14.950	2.011-111.151
Disease	Trauma vs Gynaecological	0.3228	3.357	0.304-37.051

AC=Arm-circumference; AFA=Arm fat-area; AMA=Arm muscle-area; CI=Confidence interval; DTD=Digestive tract diseases; HEI/ER <75%=Habitual energy intake <75% of the energy requirement; P=Percentile

Mirmiran *et al.* (23) found that 22.4% of the patients who lost ≥5% of their body-weights in the month before admission and 3.1% of those who lost 5 to 10% of their body-weights 3 to 6 months before admission had low energy intake.

The present sample represents most hospitalized surgical patients well. BMI, if sufficiently sensitive, could be a good indicator of patients that require special care. The BMIs of patients with digestive tract diseases and neoplasms were very good indicators of nutritional status. In general, patients with neoplasms have the highest prevalence of malnutrition, and the relative risk of death doubles in malnourished patients (30,31).

The high proportion of patients with recent weight loss (20.7%) corroborates the findfings of Caccialanza *et al* (6) who found a recent prevalence of 22.8% weight loss in hospitalized patients. These proportions are within those reported in the literature, which vary from 3.2% in orthopaedic and thoracic surgery patients (32) to approximately 39% in all types of patients (23).

Assessment of nutritional status based on BMI, recent weight loss, and low energy intake has already been made by other studies with hospitalized (9,12), pre-operative and post-operative patients (32). A multicentric study that assessed nutritional status and clinical outcomes found an HEI/ER <75% rate of 32.4% (24).

The present study found that malnutrition was significantly associated with old age, neoplasms, digestive tract diseases, head and neck surgeries, longer stays at the hospitals, number of drugs prescribed during hospital stay, recent weight loss, and the body-composition parameters. The number of medications prescribed during stay at the hospital, old age, and malignancy have been reported as independent risk factors of malnutrition (5). In the present study, malnutrition was not associated with the presence and type of complications, haemoglobin level, energy intake, and death. One study found that higher risk of morbidity and mortality was not associated with recent weight loss, hypo-albuminaemia, and low BMI in surgical gastric cancer patients (13), and another study found a nutritional risk prevalence of 14.3% in surgical patients, and malnourished patients were three times more likely to experience complications and required significantly longer hospital stays than nourished patients (10 vs 4 days, p<0.001) (12). The patients treated in the study hospital probably had a low socioeconomic status, which might have affected their nutritional status.

**Table 5.** Risk factors associated with complications according to univariate and multiple Cox's regression

Univariate analysis				
Variable	Reference	p value	Relative risk	CI (95%)
Gender	Male vs Female	0.8379	1.052	0.646-1.714
Age-group	60-69 vs <60 years	0.0812	1.744	0.933-3.259
Age-group	70-79 vs <60 years	0.0456	1.962	1.013-3.799
Age-group	≥80 vs <60 years	0.0302	2.617	1.097-6.245
Age		0.0006	1.026	1.011-1.041
Disease	DTD vs Gynaecological	0.3787	1.448	0.635-3.302
Disease	Vascular vs Gynaecological	0.6127	1.283	0.488-3.371
Disease	Neoplasms vs Gynaecological	0.0482	2.071	1.006-4.263
Disease	Trauma vs Gynaecological	0.7893	1.141	0.434-2.997
Malnourished	Yes vs No	0.3774	1.315	0.716-2.414
AC	≤P5 vs >P15	0.4272	1.307	0.675-2.530
AC	P5-P15 vs >P15	0.9352	1.030	0.503-2.110
TST	≤P5 vs >P15	0.9798	1.015	0.318-3.245
TST	P5-P15 vs >P15	0.7018	1.179	0.508-2.738
AMC	≤P5 vs >P15	0.2284	1.397	0.811-2.407
AMC	P5-P15 vs >P15	0.3693	0.691	0.308-1.549
AMA	≤P5 vs >P15	0.9071	1.035	0.578-1.854
AMA	P5-P15 vs >P15	0.0482	0.240	0.058-0.989
AFA	≤P5 vs >P15	0.8893	1.068	0.421-2.711
AFA	P5-P15 vs >P15	0.8237	1.175	0.284-4.867
Haemoglobin		0.0833	0.918	0.832-1.011
HEI/ER <75%		0.2758	0.995	0.987-1.004
Prescriptions		0.9472	1.003	0.927-1.084
Lymphocytes		0.8935	1.000	1.000-1.000
BMI		0.0908	1.040	0.994-1.088
Multiple analysis			
n=33 vs n=215				
Age		0.0114	1.032	1.007-1.058
BMI		0.0364	1.066	1.004-1.132

AC=Arm-circumference; AFA=Arm fat-area; AMA=Arm muscle-area; AMC=Arm muscle-circumference; BMI=Body mass index; CI=Confidence interval; DTD=Digestive tract diseases; HEI/ER <75%=Habitual energy intake below 75% of the energy requirement; P=Percentile; TST=Triceps skinfold thickness

According to multiple regression analysis, the most important determinants of malnutrition were age >70 years, digestive tract diseases, and neoplasms. The other study variables were not associated with malnutrition. Other studies using multiple regression analyses found that risk of malnutrition was positively correlated with old age, recent weight loss, and malignant diseases (33). Marco *et al.* (10) found that all variables in their study were independently associated with malnutrition, especially dementia, HIV infection, and pressure ulcers. The present findings indicate the importance of making a nutritional diagnosis, in addition to the clinical diagnosis, shortly after admission.

Like the present study, other studies also found that older patients (30) and those with longer stays at the hospital (24) were more vulnerable to complications. However, unlike the present study, other studies found an increased risk of complications in patients with recent weight loss (30). The small number of patients with complications in the present study may justify this fact. Nevertheless, other studies (13,32) analyzed nutritional status, post-operative complications, and predictors of surgery-related infections but also failed to find an association between recent weight loss and complications. Finally, a study found that complications were strongly associated with disease severity and nutritional status, but not with age >70 years (12).

No association was found between malnutrition and complications. On the other hand, Schiesser *et al*. (12) found that complication rates were significantly higher in patients at nutritional risk: 40% of those at nutritional risk versus 15% of those without nutritional risk experienced complications (p<0.001); they also found a high prevalence of nutritional risk in patients with gastrointestinal surgery. Multiple regression analyses showed that post-operative complications correlated positively with pancreatic surgery, old age, recent weight loss, low serum albumin, and infrequent nutritional support, which corroborated findings from other studies (30).

The other study variables did not affect the complication odds during hospital stay. However, multiple regression analysis showed that age and BMI were determinants of complications. Age and BMI differed significantly in the multiple regression analyses. Therefore, nutritional status based on BMI and old age was independently associated with complications. Old age may compromise metabolism and catabolism, resulting in lower BMI and (multi)organ failure. Vitamin and other micronutrient deficiencies were also common.

The findings of this study reinforce the importance of assessing the nutritional status right after admission. These also indicate the need for developing and implementing protocols for nutritional screening, care, diagnosis, and monitoring during stay at the hospital. These protocols would enable the proposition of intervention strategies to improving patients’ clinical courses.

### Limitations

This study has some limitations. Nutritional status was classified according to BMI, AMC, and TST (2,26). Although anthropometric parameters are considered pertinent to the nutritional status classification of hospitalized surgical patients, BMI can be an insensitive indicator because it does not reflect acute malnutrition, such as involuntary weight loss. The present study looked into recent weight loss but did not include it in the classification of nutritional status. Other limitations include not investigating the patients’ blood sugar levels, socioeconomic and behavioural characteristics, duration of disease, and treatment.

### Conclusions

The risk of malnutrition is associated with age and type of disease; old age and low BMI may promote complications.

## ACKNOWLEDGEMENTS

The study was supported by the Research Support Fund of the Pontifical Catholic University of Campinas-SP-Brazil (PUC-Campinas-SP-Brazil).
